# Use patterns of cigarettes and alternative tobacco products and socioeconomic correlates in Hong Kong secondary school students

**DOI:** 10.1038/s41598-021-96452-y

**Published:** 2021-08-26

**Authors:** Lijun Wang, Jianjiu Chen, Lok Tung Leung, Sai Yin Ho, Tai Hing Lam, Man Ping Wang

**Affiliations:** 1 School of Public Health, University of Hong Kong, Hong Kong, People’s Republic of China; 2 Department of Epidemiology, Mailman School of Public Health, Columbia University, New York, NY USA; 3 School of Nursing, University of Hong Kong, Hong Kong, People’s Republic of China

**Keywords:** Risk factors, Epidemiology, Health policy, Public health

## Abstract

Smoking is a major cause of health inequities. However, sociodemographic differences in adolescent tobacco use are unclear. In a territory-wide school-based anonymous survey in 2018/19, we investigated tobacco use and sociodemographic correlates in 33,991 students (mean age 14.8 ± 1.9 years) in Hong Kong. Tobacco use prevalence and current-ever use ratios by sociodemographic factors were calculated. Generalised linear mixed models were used in association analyses. Current use was highest for cigarettes (3.2%), closely followed by alternative tobacco products (3.0%). Current-ever use ratios were highest for heated tobacco products (HTPs, 0.60), followed by nicotine e-cigarettes (0.52), waterpipe (0.51), and cigarettes (0.35). Use prevalence and current-ever use ratios of all products showed curvilinear relations with perceived family affluence (*P* values < 0.01), being highest in the richest families. Tobacco use was also associated with more senior grades, the lowest parental education, and boys, but current-ever use ratios of HTPs and waterpipe were higher in girls (*P* values < 0.05). The results suggested that adolescent ever users of nicotine-containing alternative tobacco products were more likely to keep using them than cigarettes, and the richest adolescents were at the highest risks of tobacco use. Diverse tobacco control measures are needed to improve health equity, especially on alternative tobacco products.

## Introduction

Smoking is a major cause of health inequities. Over 80% of smokers live in low- and middle-income countries (LMIC), where over 80% of smoking-related deaths were predicted to occur by 2030^1,2^. The inequities are also evident within countries and regions, showing inverse associations between smoking and socioeconomic status (SES) in some developed countries^3,4^. Disadvantaged people tend to initiate smoking at younger ages, smoke heavier, and be less successful in quitting^4^. Diverting their limited disposable income to tobacco means lower expenditures on food, shelter, education, and healthcare^5,6^, aggravating socioeconomic inequities.

However, studies have shown inconsistent associations between family SES and adolescent cigarette smoking. In the United States (US) and the United Kingdom (UK), lower family SES strongly predicted adolescent smoking initiation and escalation^7–9^. A study in 1,308 US adolescents showed that each level lower in household income and parental education was associated with 30% and 28% higher risks of adolescent smoking, respectively^9^. In contrast, the World Health Organisation (WHO) Health Behaviour in School-aged Children (HBSC) study in 35 Western countries showed that each standard deviation (SD) lower family affluence was only associated with a modest (9%) increase in the odds of adolescent weekly smoking^10^. The associations were generally stronger in more affluent countries, and non-significant in over half the countries in the HBSC study. Studies in developing countries and regions at relatively early stages of the smoking epidemic are scarce. Associations similar to those in the US and UK were reported in mainland China^11^, but no association was found in Ghana^12^.

The associations between SES and use of alternative tobacco products remain unclear in adolescents. Studies in the US and New Zealand have found higher e-cigarette (EC) use in adolescents from low-SES families and communities^13,14^. However, other studies showed that SES was unassociated^15–17^, or even positively associated with adolescent EC use or use susceptibility^18,19^. We found only 1 study on the correlates of heated tobacco product (HTP) use—the Korea Youth Risk Behavior Web-based Survey, which showed that compared with the highest-SES group, the prevalence of ever HTP use was 27% lower in the middle-SES group but similar in the lowest-SES group^20^. Waterpipe use was more common in youths who received the largest amount of spending money in most of the 60 countries in a secondary analysis based on the Global Youth Tobacco Survey^21^.

Current-ever use ratio (the proportion of current users among ever users) of a tobacco product is a useful indicator of how likely the product is used beyond experimentation and can be assessed using cross-sectional data. However, only 1 study had reported such current-ever use ratio, with a value of 0.27 for ECs in US adults in 2014^22^, and no relevant reports for adolescents were found up to 31 March 2021 (see search strategies in Supplementary note).

Hong Kong has implemented comprehensive tobacco control measures and achieved one of the lowest current smoking prevalence (10.8% smoking in persons aged over 15 in 2019) across the world^23^. However, the emergence of alternative tobacco products threatens to renormalise tobacco use and reverse its declining secular trend. Although the sale and import of nicotine ECs and HTPs have not been approved as of 11 April 2021^24^, they are available in online and physical stores^25^. Given that 89.1% of current smokers aged 20–54 years starting regular smoking before age 25, adolescence is regarded as a crucial window for tobacco control^26^. Therefore, the objectives of the present study were to compare the use patterns of cigarettes, ECs, HTPs, and waterpipe, and examine their associations with SES in Hong Kong secondary school students, to inform future tobacco control policies and practices.

## Methods

### Study design

The School-based Smoking Survey is a territory-wide biennial smoking survey in secondary school students (US grades 7–12) in Hong Kong. The present round of survey was conducted from October 2018 to July 2019. Details of the survey methods have been reported^27–29^. Briefly, a stratified random sample of schools in all 18 districts of Hong Kong were invited in proportion to the total number of schools in each district. Parental informed consent was obtained from all parents for participation of their children before the survey. All parents in recruited schools received an invitation letter via students, and declining parents were to ask students to return a blank answer sheet during the survey. At the beginning of the survey, class teachers explained that participation was voluntary and students could decline even with parental consent. Ethics approval was obtained from the Institutional Review Board (IRB) of the University of Hong Kong/Hospital Authority Hong Kong West Cluster. All methods were carried out in accordance with the Declaration of Helsinki and relevant regulations of IRB.

We invited all students in participated schools to complete a standardised structured questionnaire using a separate anonymous answer sheet in classrooms. Following standard survey procedures on an instruction sheet, the teachers distributed questionnaires and answer sheets, and maintained classroom order. The questionnaire took 20 min to complete, covering sociodemographic characteristics, tobacco-related knowledge and attitudes, susceptibility to and behaviours of tobacco use, secondhand smoke exposure, and other health-related items. We assigned at least 1 trained research assistant per grade to help coordinate and answer students’ queries during the survey. To encourage candid reporting, teachers avoided patrolling near students. Completed answer sheets were immediately sealed in front of the students and collected by research staff. Students who were absent received a pack with the questionnaire and answer sheet from teachers later to be returned directly to us in a prepaid envelope.

In total, 34,063 students from 88 schools participated, with student- and school-level response rates of 94% and 23%, respectively. Non-participation of schools was usually due to administrative reasons such as tight schedule rather than smoking-related issues. We used Remark Office OMR 8.0 software^30^ to capture data on the answer sheets, with a low error rate of 0.3% verified by a 5% manual data entry. Responses with over 50% missing data were excluded (0.2%), leaving 33,991 for analysis.

### Measures

We separately assessed ever use of various tobacco products with the item “Please choose one option that suits you most regarding each of the following products (cigarette/electronic cigarette/heated tobacco product/waterpipe/other tobacco products, e.g. cigar and snus)”. The options were the same for various products, including “I have never used it”, “I have used it once or a few times (for fun or to try a puff)”, “I used to use it occasionally (not every day), but have quit now”, “I used to use it every day, but have quit now”, “I use it occasionally (not every day)”, and “I use it every day”. Students were classified as never users for choosing “I have never used it”, experimenters for “I have used it once or a few times (for fun or to try a puff)”, and ever users for any options other than “I have never used it”. Current use of each tobacco product was assessed similarly by asking “On how many of the past 30 days did you use the following products (cigarette/electronic cigarette/heated tobacco product/waterpipe/other tobacco products, e.g. cigar and snus)”, with options of “0/1–2/3–5/6–9/10–19/20–29/30 days”. Current use of a product was defined as having used it for at least 1 day in the past 30 days^31^.

As non-nicotine EC products are common in Hong Kong, we also asked “Do you use electronic cigarettes containing nicotine?” Students were classified as ever nicotine EC users for choosing “Some contain nicotine” or “All contain nicotine”, never nicotine EC users for choosing “None of them contain nicotine”, and never EC users for choosing “I have never used electronic cigarettes”.

We assessed two dimensions of family SES—parental education (options: “primary or below”, “secondary”, “post-secondary”, and “don’t know”) and perceived family affluence. As income inequality in Hong Kong is among the highest in the world^32^ and easily perceivable (e.g. by type, size, and location of housing), we assessed students’ perceived family affluence with the item “You consider your family’s economic status:” (options: “relatively poor”, “poor to average”, “average”, “average to rich”, and “relatively rich”)^33,34^. We also collected students’ sociodemographic characteristics including sex, age, and school grade.

### Statistical analysis

We analysed 8 categories of tobacco products, i.e. any tobacco products, alternative tobacco products, cigarettes, ECs, nicotine ECs, non-nicotine ECs, HTPs, and waterpipe. Use of any tobacco products was defined as use of at least one category of “cigarette/electronic cigarette/heated tobacco product/waterpipe/other tobacco products (e.g. cigar and snus)”; use of alternative tobacco products was defined as use of at least one category other than “cigarette”. The use prevalence and current-ever use ratio of each category of tobacco products by sociodemographic characteristic were calculated. Current-ever use ratio was the proportion of current users among ever users. All the proportions with 95% confidence intervals were weighted by sex, age, and grade distribution of the underlying population provided by the Education Bureau of the Government of the Hong Kong Special Administrative Region (SAR).

For each specific tobacco product (cigarettes, ECs, HTPs, and waterpipe), we calculated adjusted odds ratios (AORs) of ever (versus never) use and current (versus non-current) use in all students, and current (versus non-current) use in ever users by sociodemographic factors, using generalised linear mixed models (GLMM) with a “logit” link function and random intercept accounting for school clustering effects, with R (version 4.0.0) package “lme4” (version 1.1-21). As age and grade were highly correlated, and the information of grade was complete and reliable, we adjusted for grade together with sex, perceived family affluence, and parental education in regression analyses. We did not differentiate nicotine and non-nicotine ECs in regression analyses, due to insufficient users in some subgroups. We also tested the trends of tobacco use by the sociodemographic factors: linear trends were first tested by including the sociodemographic factors as continuous variables; quadratic trends were subsequently tested by including the sociodemographic factors as continuous variables if the AORs showed curvilinear trends. *P* values of less than 0.05 were considered statistically significant. We assessed interactions between sex and other sociodemographic factors, with the reference groups consistent with those in the main analyses. We found sex interactions and reported stratified results for boys and girls. We conducted two sensitivity analyses: (1) current-ever use ratios were calculated excluding experimenters to avoid classifying recent experimenters as current users; (2) students who had never used any tobacco products instead of just the specific tobacco product being analysed were treated as the reference group^35^.

## Results

The sample had a mean age (SD) of 14.8 (1.9) years. Table 1 shows that 51.5% students were boys, and 13.1% had ever used any tobacco products, with cigarettes (9.1%) being the most common, followed by ECs (7.9%), waterpipe (3.6%), and HTPs (2.6%). Only 4.1% were current users of any tobacco products. The current-ever use ratios were higher for HTPs (0.60), nicotine ECs (0.52), and waterpipe (0.51), but lower for cigarettes (0.35) and non-nicotine ECs (0.22). Current-ever use ratios were highest for HTP in girls (0.68). Confidence intervals of the weighted prevalence are shown in Supplementary Table S1.

**Table 1:** Use of various tobacco products in Hong Kong secondary school students by sociodemographic factors.

	All (%), n = 33,991	Ever use (%)								Current use (%)							
		Any TPs	Alt TPs	Cig	EC	N-Nct EC	Nct EC	HTP	WP	Any TPs	Alt TPs	Cig	EC	N-Nct EC	Nct EC	HTP	WP
**Overall**	–	13.1	9.2	9.1	7.9	5.6	2.3	2.6	3.6	4.1	3.0	3.2	2.5	1.3	1.2	1.6	1.8
**Sex**																	
Boys	51.5	14.9	10.3	10.6	8.9	6.3	2.6	3.1	4.1	4.6	3.3	3.6	2.8	1.5	1.3	1.7	2.0
Girls	48.6	11.2	8.0	7.6	6.9	4.8	2.0	2.0	3.0	3.6	2.6	2.8	2.2	1.1	1.1	1.4	1.7
**Age**																	
≤ 12	13.4	6.0	3.7	4.2	3.1	2.2	0.9	1.5	1.6	2.3	2.0	1.8	1.7	1.0	0.7	1.2	1.2
13	16.2	7.5	4.9	5.2	4.2	3.0	1.2	1.5	1.5	2.4	1.8	1.7	1.5	0.9	0.6	0.9	0.9
14	16.3	10.7	6.7	7.5	5.7	4.1	1.7	1.4	1.6	2.9	1.9	2.2	1.7	1.0	0.7	0.8	0.9
15	15.2	13.3	9.9	8.5	8.8	6.2	2.7	2.3	2.9	4.3	3.3	3.2	2.8	1.2	1.6	1.8	2.0
16	16.3	15.1	10.6	10.3	9.1	6.4	2.6	2.8	3.7	4.4	3.1	3.5	2.6	1.3	1.3	1.8	2.0
17	15.9	18.7	14.2	12.6	12.2	8.8	3.4	3.6	6.4	5.5	3.8	4.3	3.1	1.5	1.7	1.8	2.6
≥ 18	6.8	27.9	19.7	22.6	16.5	11.0	5.5	7.6	11.4	10.6	7.7	8.5	5.9	3.1	2.9	4.1	5.0
**Grade**																	
7	18.0	6.6	3.9	4.6	3.2	2.2	0.9	1.1	1.1	1.9	1.5	1.3	1.2	0.7	0.5	0.8	0.8
8	17.2	9.3	5.9	6.6	5.1	3.5	1.6	1.6	1.8	2.9	1.9	2.3	1.7	1.1	0.6	1.0	1.0
9	16.6	11.6	7.7	8.1	6.7	4.8	2.0	1.8	2.1	3.6	2.5	2.8	2.0	1.1	0.9	0.9	1.1
10	16.3	15.6	11.4	10.6	10.0	7.1	2.9	2.9	3.9	5.1	3.6	4.0	3.2	1.5	1.7	2.0	2.3
11	16.0	16.5	11.9	11.2	10.1	7.3	2.9	3.5	4.8	4.9	3.6	3.7	3.1	1.6	1.5	2.1	2.6
12	15.9	20.1	15.4	14.4	13.2	9.1	4.1	4.6	8.1	6.6	4.9	5.3	4.0	1.8	2.2	2.6	3.4
**Perceived family affluence**																	
Relatively poor	5.7	19.4	12.9	15.5	11.7	8.2	3.5	5.6	6.6	7.1	5.3	5.7	4.6	2.6	2.1	3.8	3.8
Poor to average	21.4	16.2	10.4	11.6	9.0	6.7	2.3	2.4	3.2	3.5	2.3	2.8	1.9	1.0	0.9	1.2	1.4
Average	58.7	11.2	8.0	7.6	6.9	4.8	2.0	1.9	2.8	3.5	2.4	2.7	2.0	1.0	1.0	1.1	1.4
Average to rich	12.4	12.0	9.3	7.9	7.6	4.9	2.7	3.1	4.6	5.2	4.1	3.5	3.3	1.7	1.7	1.9	2.4
Relatively rich	1.8	23.4	21.0	18.4	19.3	11.9	7.4	12.8	14.6	15.8	14.4	13.3	12.4	6.5	5.9	9.1	10.2
**Parental education**																	
Primary or below	5.0	20.5	14.1	15.2	12.6	9.3	3.4	4.5	5.3	6.2	4.7	4.9	3.9	2.4	1.5	2.8	3.0
Secondary	50.4	14.2	9.4	10.0	8.2	6.0	2.2	2.0	3.0	3.6	2.3	2.9	1.9	1.0	0.8	1.1	1.4
Tertiary	26.7	10.2	8.2	6.7	6.8	4.2	2.6	2.9	4.1	4.5	3.9	3.1	3.3	1.4	1.9	2.0	2.3
Unknown	17.9	12.0	8.5	8.4	7.4	5.4	2.1	2.9	3.5	4.5	3.0	3.7	2.5	1.4	1.2	1.8	2.0

	Current-ever use ratio																
	Any TPs	Alt TPs	Cig	EC	N-Nct EC	Nct EC	HTP	WP									
Overall	0.31	0.32	0.35	0.31	0.22	0.52	0.60	0.51									
**Sex**																	
Boys	0.31	0.31	0.33	0.31	0.23	0.50	0.55	0.47									
Girls	0.32	0.33	0.37	0.32	0.22	0.54	0.68	0.56									
**Age**																	
≤ 12	0.37	0.51	0.41	0.53	0.43	0.73	0.78	0.74									
13	0.32	0.37	0.33	0.35	0.30	0.49	0.61	0.59									
14	0.27	0.28	0.29	0.29	0.23	0.41	0.55	0.50									
15	0.32	0.33	0.38	0.32	0.20	0.60	0.74	0.68									
16	0.29	0.29	0.34	0.29	0.20	0.50	0.66	0.55									
17	0.29	0.26	0.34	0.25	0.16	0.48	0.49	0.40									
≥ 18	0.38	0.39	0.37	0.36	0.28	0.52	0.54	0.44									
**Grade**																	
7	0.29	0.38	0.27	0.38	0.32	0.51	0.72	0.70									
8	0.31	0.33	0.35	0.33	0.29	0.40	0.61	0.53									
9	0.31	0.32	0.35	0.30	0.23	0.46	0.52	0.49									
10	0.33	0.32	0.38	0.32	0.21	0.59	0.69	0.59									
11	0.30	0.31	0.33	0.30	0.22	0.51	0.60	0.55									
12	0.33	0.32	0.36	0.30	0.19	0.55	0.55	0.42									
**Perceived family affluence**																	
Relatively poor	0.36	0.40	0.36	0.39	0.30	0.60	0.64	0.56									
Poor to average	0.21	0.22	0.24	0.21	0.15	0.40	0.50	0.44									
Average	0.31	0.30	0.35	0.29	0.20	0.49	0.60	0.49									
Average to rich	0.43	0.44	0.45	0.44	0.34	0.62	0.62	0.52									
Relatively rich	0.67	0.68	0.72	0.64	0.54	0.80	0.71	0.69									
**Parental education**																	
Primary or below	0.30	0.32	0.31	0.30	0.23	0.46	0.58	0.56									
Secondary	0.25	0.24	0.28	0.23	0.17	0.38	0.54	0.44									
Tertiary	0.44	0.47	0.47	0.49	0.34	0.72	0.67	0.56									
Unknown	0.37	0.35	0.44	0.34	0.25	0.55	0.63	0.55									

All percentages were weighted by sex, age, and grade distribution of the underlying population provided by the Education Bureau of the Hong Kong Special Administrative Region Government.

TP, tobacco product; Alt TP, alternative tobacco product; Cig, cigarette; EC, e-cigarette; N-Nct, non-nicotine; Nct, nicotine; HTP, heated tobacco product; WP, waterpipe.

**Figure 1:**
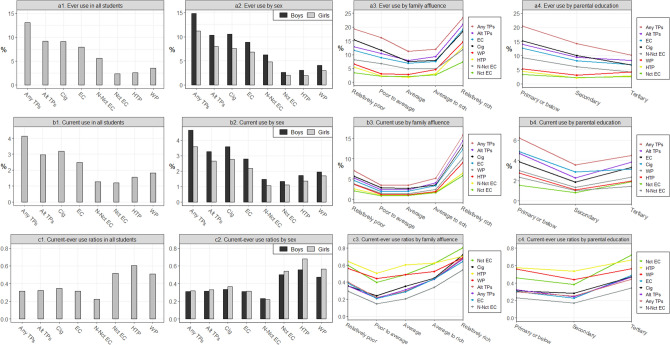
Use of various tobacco products in Hong Kong secondary school students by sociodemographic factors. TP, tobacco product; Alt TP, alternative tobacco product; Cig, cigarette; EC, e-cigarette; N-Nct, non-nicotine; Nct, nicotine; HTP, heated tobacco product; WP, waterpipe. *Note*: In line graphs, the position of legends was aligned with the rank of the last value on each line. All percentages were weighted by sex, age, and grade distribution of the target population provided by the Education Bureau of the Government of the Hong Kong Special Administrative Region.

Figure 1 a1–c4 depicts use patterns of various tobacco products by sociodemographic factors. The most commonly used category was cigarettes in the poorest families, but alternative tobacco products in the richest families (ever use in Fig. 1 a3 and current use in Fig. 1 b3). Ever use prevalence, current use prevalence, and current-ever use ratios (Fig. 1 a3,b3,c3) of all categories showed J-shaped relations with perceived family affluence, being highest in students from the richest families, followed by the poorest families and the middle groups. Similar patterns were observed for current use prevalence and current-ever use ratios by parental education (Fig. 1 b4,c4).

Table 2 shows that ever use of the 4 products was more common in boys and increased with grade (*P* values < 0.01). The AORs of ever use of the 4 products showed curvilinear relations with perceived family affluence (*P* values < 0.001), being lowest in the middle groups and highest in the richest groups. The AORs of ever use of HTPs and waterpipe showed curvilinear relations with parental education (*P* values < 0.001), while those of cigarettes and ECs decreased with parental education (*P* values < 0.01). Table 3 shows that current cigarette use was less common in girls (AOR 0.86, 95% CI 0.75–0.98). Current use of the 4 products was associated with more senior grades (*P* values < 0.001). The AORs of current use of the 4 products showed curvilinear relations with perceived family affluence and parental education (*P* values < 0.01). Table 4 shows that current use in ever users for HTPs (AOR 1.44, 95% CI 1.04–1.97) and waterpipe (AOR 1.50, 95% CI 1.14–1.96) were more common in girls than in boys. No clear trends by grade were observed for current use in ever users of ECs, HTPs, and waterpipe. The AORs of current use in ever users of the 4 products showed curvilinear relations with perceived family affluence and parental education (*P* values < 0.05).

**Table 2:** Associations between sociodemographic factors and ever (vs never) tobacco use in Hong Kong secondary school students.

	AOR (95% CI)^a^			
	Cigarette	EC	HTP	Waterpipe
**Sex**				
Boys	1	1	1	1
Girls	0.78 (0.71, 0.84)***	0.86 (0.79, 0.94)***	0.75 (0.65, 0.87)***	0.80 (0.70, 0.92)**
**Grade**				
7	1	1	1	1
8	1.47 (1.26, 1.71)***	1.55 (1.30, 1.85)***	1.37 (1.02, 1.83)*	1.53 (1.15, 2.04)**
9	1.79 (1.54, 2.07)***	2.04 (1.72, 2.42)***	1.60 (1.20, 2.12)**	1.92 (1.46, 2.53)***
10	2.26 (1.95, 2.61)***	2.99 (2.54, 3.53)***	2.23 (1.70, 2.93)***	3.11 (2.39, 4.05)***
11	2.28 (1.96, 2.65)***	2.96 (2.50, 3.51)***	2.68 (2.04, 3.52)***	3.79 (2.91, 4.93)***
12	2.93 (2.46, 3.50)***	3.81 (3.14, 4.62)***	3.47 (2.54, 4.74)***	6.60 (4.95, 8.80)***
P for trend	< 0.001^**†**^	< 0.001^**†**^	< 0.001^**†**^	< 0.001^**†**^
**Perceived family affluence**				
Relatively poor	1	1	1	1
Poor to average	0.77 (0.67, 0.89)***	0.77 (0.66, 0.91)**	0.41 (0.32, 0.52)***	0.44 (0.35, 0.55)***
Average	0.60 (0.52, 0.69)***	0.72 (0.61, 0.83)***	0.40 (0.32, 0.49)***	0.45 (0.37, 0.55)**
Average to rich	0.76 (0.63, 0.90)**	0.91 (0.75, 1.10)	0.69 (0.52, 0.90)**	0.77 (0.60, 0.98)*
Relatively rich	1.67 (1.30, 2.14)***	2.26 (1.74, 2.92)***	2.59 (1.87, 3.58)***^§^	2.65 (1.95, 3.61)***
P for trend	< 0.001^‡^	< 0.001^‡^	< 0.001^‡^	< 0.001^‡^
**Parental education**				
Primary or below	1	1	1	1
Secondary	0.82 (0.71, 0.95)**	0.80 (0.68, 0.94)**	0.61 (0.47, 0.78)*	0.70 (0.55, 0.88)**
Tertiary	0.64 (0.54, 0.75)***	0.70 (0.58, 0.84)***	0.80 (0.61, 1.06)	0.77 (0.60, 1.00)*
P for trend	< 0.001^**†**^	< 0.01^**†**^	< 0.001^‡^	< 0.001^‡^

*P < 0.05, **P < 0.01, ***P < 0.001. ^†^Linear trend. ^‡^Curvilinear (quadratic) trend. ^§^Interaction with sex. EC, e-cigarette; HTP, heated tobacco product; AOR, adjusted odds ratio; CI, confidence interval.

^a^Adjusted odd odds ratios adjusted for sex, grade, perceived family affluence, parental education, and school clustering effects.

**Table 3:** Associations between sociodemographic factors and current (vs non-current) tobacco use in Hong Kong secondary school students.

	AOR (95% CI)^a^			
	Cigarette	EC	HTP	Waterpipe
**Sex**				
Boys	1	1	1	1
Girls	0.86 (0.75, 0.98)*	0.88 (0.76, 1.02)	0.92 (0.77, 1.11)	0.97 (0.82, 1.16)
**Grade**				
7	1	1	1	1
8	1.80 (1.38, 2.33)***	1.32 (0.99, 1.76)	1.18 (0.83, 1.68)	1.24 (0.87, 1.77)
9	2.19 (1.70, 2.83)***	1.58 (1.19, 2.08)**	1.30 (0.92, 1.83)	1.54 (1.10, 2.17)*
10	2.85 (2.23, 3.66)***^§^	2.14 (1.64, 2.81)***	2.12 (1.54, 2.93)***	2.49 (1.81, 3.44)***
11	2.50 (1.93, 3.23)***	2.05 (1.55, 2.70)***	2.22 (1.60, 3.09)***	2.86 (2.07, 3.95)***
12	3.22 (2.40, 4.31)***	2.44 (1.77, 3.36)***	2.50 (1.70, 3.67)***^§^	3.68 (2.54, 5.33)***^§^
P for trend	< 0.001^**†**^	< 0.001^**†**^	< 0.001^**†**^	< 0.001^**†**^
**Perceived family affluence**				
Relatively poor	1	1	1	1
Poor to average	0.53 (0.42, 0.67)***	0.40 (0.31, 0.52)***	0.31 (0.23, 0.41)***	0.35 (0.26, 0.47)***
Average	0.58 (0.47, 0.72)***	0.45 (0.36, 0.57)***	0.33 (0.25, 0.43)***	0.36 (0.28, 0.46)***
Average to rich	0.89 (0.69, 1.16)	0.76 (0.58, 1.01)	0.63 (0.45, 0.87)**	0.67 (0.49, 0.93)*
Relatively rich	3.09 (2.25, 4.24)***	2.75 (1.97, 3.85)***^§^	2.91 (2.02, 4.19)***^§^	3.16 (2.21, 4.52)***^§^
P for trend	< 0.001^‡^	< 0.001^‡^	< 0.001^‡^	< 0.001^‡^
**Parental education**				
Primary or below	1	1	1	1
Secondary	0.66 (0.53, 0.83)***	0.53 (0.41, 0.69)***	0.43 (0.32, 0.57)***	0.49 (0.37, 0.65)***
Tertiary	0.69 (0.54, 0.90)**	0.70 (0.53, 0.94)*	0.63 (0.46, 0.88)**^§^	0.68 (0.49, 0.93)*^§^
P for trend	< 0.01^‡^	< 0.001^‡^	< 0.001^‡^	< 0.001^‡^

*P < 0.05, **P < 0.01, ***P < 0.001. ^†^Linear trend. ^‡^Curvilinear (quadratic) trend. ^§^Interaction with sex. EC, e-cigarette; HTP, heated tobacco product; AOR, adjusted odds ratio; CI, confidence interval.

^a^Adjusted odds ratios adjusted for sex, grade, perceived family affluence, parental education, and school clustering effects.

**Table 4:** Associations between sociodemographic factors and current (vs non-current) tobacco use in ever users.

	AOR (95% CI)^a^			
	Cigarette	EC	HTP	Waterpipe
**Sex**				
Boys	1	1	1	1
Girls	1.00 (0.84, 1.19)	0.91 (0.75, 1.10)	1.44 (1.04, 1.97)*	1.50 (1.14, 1.96)**
**Grade**				
7	1	1	1	1
8	1.19 (0.82, 1.71)	0.77 (0.52, 1.14)	0.69 (0.36, 1.32)	0.58 (0.31, 1.08)
9	1.47 (1.04, 2.09)*	0.81 (0.56, 1.19)	0.71 (0.38, 1.32)	0.70 (0.38, 1.26)
10	1.81 (1.29, 2.54)***	0.78 (0.54, 1.12)	1.12 (0.62, 2.02)	0.77 (0.44, 1.35)
11	1.50 (1.06, 2.13)*	0.81 (0.56, 1.18)	0.85 (0.47, 1.53)	0.71 (0.41, 1.25)
12	1.71 (1.15, 2.52)**	0.73 (0.48, 1.11)	0.63 (0.33, 1.20)^§^	0.51 (0.28, 0.92)*^§^
P for trend	0.04^**†**^	0.41^**†**^	0.18^**†**^	0.052^**†**^
**Perceived family affluence**				
Relatively poor	1	1	1	1
Poor to average	0.59 (0.44, 0.80)***	0.39 (0.27, 0.55)***	0.44 (0.26, 0.74)**	0.55 (0.34, 0.88)*
Average	0.90 (0.68, 1.19)	0.54 (0.39, 0.73)***	0.60 (0.37, 0.97)*	0.65 (0.43, 0.99)*
Average to rich	1.44 (1.02, 2.05)*	0.97 (0.67, 1.42)	0.88 (0.50, 1.57)	0.85 (0.51, 1.40)
Relatively rich	4.40 (2.70, 7.16)***	1.99 (1.21, 3.26)**^§^	1.82 (0.89, 3.73)^§^	1.80 (0.96, 3.38)
P for trend	< 0.001^‡^	< 0.001^‡^	< 0.001^‡^	< 0.01^‡^
**Parental education**				
Primary or below	1	1	1	1
Secondary	0.64 (0.48, 0.86)**	0.59 (0.42, 0.83)**	0.56 (0.31, 0.98)*	0.55 (0.33, 0.89)*
Tertiary	1.13 (0.81, 1.59)^§^	1.10 (0.76, 1.59)^§^	0.74 (0.40, 1.35)	0.83 (0.49, 1.40)
P for trend	< 0.001^‡^	< 0.001^‡^	0.04^‡^	< 0.01^‡^

*P < 0.05, **P < 0.01, ***P < 0.001. ^†^Linear trend. ^‡^Curvilinear (quadratic) trend. ^§^Interaction with sex. EC, e-cigarette; HTP, heated tobacco product; AOR, adjusted odds ratio; CI, confidence interval.

^a^Adjusted odds ratios adjusted for sex, grade, perceived family affluence, parental education, and school clustering effects.

Only a few levels of the sociodemographic factors had interaction effects with sex (Tables 2, 3, and 4), and no marked differences were observed in the patterns of AORs between sexes (Supplementary Table S2). The first sensitivity analysis showed that, after excluding experimenters, the current-ever use ratios increased for each tobacco product, but the ratios for HTPs (0.88) and waterpipe (0.84) still appeared to be higher than that of cigarettes (0.77) (Supplementary Table S3). The second sensitivity analysis showed that, with students who had never used any tobacco products as the reference group, the sociodemographic differences (AORs) between users and non-users of specific tobacco products were generally unchanged (Supplementary Table S4). These sensitivity analyses showed the robustness of the results from the main analyses.

## Discussion

We have first used the current-ever use ratio as an indicator of how likely a tobacco product is used beyond experimentation among adolescents using cross-sectional data. The results suggested that ever users of nicotine-containing alternative tobacco products were more likely to keep using them than ever users of cigarettes and non-nicotine products. Despite the lower use prevalence in girls, they were more likely than boys to keep using waterpipe and HTPs among ever users. In addition, we showed the socioeconomic gradient in users of various tobacco products, and found J-shaped relations that students from the richest families had the highest use prevalence and the middle groups had the lowest use prevalence. Moreover, the most common category of tobacco product for both ever and current use was cigarettes in the poorest families, but alternative tobacco products in the richest families.

The higher prevalence of tobacco use in students from the poorest families than middle-SES families could be due to parental smoking, peer influence, single parents, stress, and poor school performance^4,7–11,36^. However, compared with students from the poorest families, higher proportions of those from the richest families used the 4 tobacco products, especially alternative tobacco products, which are more expensive (a pack of cigarettes cost US$7.1–9.0 including US$4.9 of tax; ECs and HTPs cost US$25–130 per reusable device set in Hong Kong). Our previous study also showed that waterpipe use was more common in Hong Kong secondary school students from rich families^37^. The affordability of tobacco products could modify the associations between family affluence and tobacco use in adolescents^10,38^, which was also shown in overseas studies. Adolescents from poor families had higher smoking prevalence in countries and regions where cigarettes were more affordable, such as mainland China (as low as US$0.5/pack)^11^ and some developed countries^7–9^, but the differences were smaller in lower-income countries^10,12^. In Ghana, smokeless tobacco (tawa) that is cheaper and more readily available than cigarettes, was more commonly used by adolescents from poor families, but cigarette use did not vary by family affluence^12^. Adolescent EC users in Korea usually had more spending money^39^. High-SES adolescents usually used JUUL rather than cheaper EC devices in the US^40^.

Apart from affordability, several reasons could help explain why alternative tobacco products were more commonly used in the most well-off adolescents. First, alternative tobacco products have targeted the middle and upper classes, touting the so-called “premium” and “high-end” lifestyle^41^. Our previous study showed that 16.6% of Hong Kong secondary school students were exposed to EC advertising in the past month mainly via online media and point-of sale marketing, and exposed students were more susceptible to EC use, suggesting that the marketing may influence their tobacco use^29^. Second, privileged social groups are usually exposed to more advertising and they try new products earlier. In England, ECs were first used by high-SES groups before spreading to low-SES groups from 2014 to 2017^42^. Third, adolescents and parents from the most affluent families would also be more health conscious and ready to pay a premium for alternative tobacco products that are claimed to be less harmful than cigarettes^25,43–45^, especially in Hong Kong, where three-quarters of people considered themselves to be health conscious^46^.

We found that current-ever use ratios were lower for cigarettes (0.35) and non-nicotine ECs (0.22), and higher for nicotine-containing alternative tobacco products—HTPs (0.60), nicotine ECs (0.52), and waterpipe (0.51). Based on the results of the National Youth Tobacco Survey (NYTS) 2019 in US students (grades 7–12)^47^, the current-ever use ratio calculated by us was 0.57 for ECs and 0.26 for cigarettes. The current-ever use ratio of nicotine ECs in Hong Kong appeared to be similar to that of ECs in the US, where almost all ECs (99%) contain nicotine^48^. High nicotine concentrations in products including JUUL and IQOS expose youth to high risk of addiction^49,50^. An adult study also showed that HTPs could reinforce nicotine dependence, rather than serving as a cessation aid^51^. In addition, child-friendly flavours of alternative tobacco products can improve use experience and reduce bitterness and harshness, which may encourage regular use^52^. We found higher current-ever use ratios of nicotine-containing alternative tobacco products in girls than boys, despite their lower prevalence of use. Compared with men, women were more health-conscious and preferred healthier products^53,54^, and thus more likely to keep using tobacco products which were perceived to be less harmful than cigarettes, such as ECs, HTPs, and waterpipe^55,56^. Prior studies also showed that women and teenage girls were more prone to nicotine addiction after smoking initiation^57^, and had more difficulties in quitting^58^. Tobacco companies have targeted women and girls using themes of beauty, fashion, freedom and sophistication, especially those in low- and middle-income countries with lower smoking rates^59,60^, and our results on their higher current-ever use ratios in girls suggest that nicotine-containing alternative tobacco products are more successful than cigarettes in this regard. On the other hand, researchers and tobacco control advocates should pay close attention to alternative tobacco product use in adolescents, considering that cigarette advertising has exploited their desire to be popular, attractive, adventurous, and mature^61^. Persistent use in ever users of non-nicotine EC products should also be monitored.

Given the J-shaped relations between perceived family affluence and various tobacco use, we propose to adopt diverse tobacco control measures to improve health equity. Raising tobacco taxes and prices is regarded as the most effective in curbing tobacco use in adolescents and disadvantaged groups^62^. The tobacco tax (US$4.9 per pack) in Hong Kong constitutes 63% of the retail price of popular cigarette brands (US$7.7 per pack), which is lower than that of at least 75% based on the WHO “MPOWER” policy package. Tobacco tax in Hong Kong has not been raised since February 2014. A 100% increase in tobacco tax has been advocated by the Hong Kong Council on Smoking and Health^63^, which will increase the amount of tax to about 77.6% of the retail price. Despite the ban on advertising, promotion and sponsorship of all tobacco products, strict enforcement for alternative tobacco products is needed in Hong Kong, especially in youth-oriented new media. In addition, health education in schools should emphasise the harms of alternative tobacco products, particularly those in high-SES districts. The government has proposed a total ban of ECs and HTPs in February 2019, but it encounters strong resistance from the tobacco industry and some pro-industry legislators. The bills are still under debate as of 10 June 2021. Our results support banning ECs and HTPs in Hong Kong to protect adolescents.

This study had some limitations. First, because our study was cross-sectional, the temporal sequence was uncertain. However, the risk factors (family SES) were unlikely to have been influenced by the outcomes (student tobacco use). Second, because HTPs were relatively new in Hong Kong, some respondents might have mistaken them for other tobacco products despite the brief introduction of HTPs in the beginning of the questionnaire. We did not mention any brands, e.g. IQOS, to avoid propaganda effects among minors. Nevertheless, respondents who had used HTPs should be able to report so. Third, the higher current-ever use ratios of alternative tobacco products might be partly due to their shorter history so that recent trial use would be more likely. The latest product included in this study was HTP, which became available and popular in Hong Kong only 1–2 years before this survey. However, the sensitivity analyses showed that, after excluding experimenters, current-ever use ratios still appeared to be higher for HTPs and waterpipe than cigarettes. Despite these results, further studies are warranted to keep monitoring the use patterns of alternative tobacco products.

Cigarette use is a major cause for health inequity, but the worldwide emergence and popularity of alternative tobacco products may alter the previous distribution of tobacco use across populations, which requires close monitoring regardless of whether the products have been legalised in the market. More stringent regulations on alternative tobacco products and comprehensive prevention measures are needed to combat tobacco renormalisation in adolescents.

## Supplementary Information

Supplementary Information.

## Data Availability

Dataset can be requested with appropriate reasons from The Food and Health Bureau, the Government of the Hong Kong Special Administrative Region. For requests, please contact the corresponding author.
