# Supplementary material for: Use patterns of cigarettes and alternative tobacco products and socioeconomic correlates in Hong Kong secondary school students
Source: Sci Rep. 2021 Aug 26;11:17253. doi: 10.1038/s41598-021-96452-y (PMC8390664; doi:10.1038/s41598-021-96452-y)
Supplement: Supplementary file 1 — Supplementary Information. [file 41598_2021_96452_MOESM1_ESM.docx]

Use patterns of cigarettes and alternative tobacco products and socioeconomic correlates in Hong Kong secondary school students

Lijun Wang, Jianjiu Chen, Lok Tung Leung, Sai Yin Ho, Tai Hing Lam and Man Ping Wang

Supplementary note.

Search strategies for publications on current-ever use ratio: searching PubMed and Web of Science using the terms of “(current ever ratio OR current ever use ratio OR current users among ever users OR current use among ever users) AND (tobacco) AND (child OR adolescent)”.

| **Supplement Table S1. Confidence intervals of the use prevalence of various tobacco products in Hong Kong secondary school students by sociodemographic factors** | | | | | | | | |
| --- | --- | --- | --- | --- | --- | --- | --- | --- |
|  | **95% Confidence Interval** | | | | | | | |
|  | **Any TPs** | **Alt TPs** | **Cig** | **EC** | **N-Nct EC** | **Nct EC** | **HTP** | **WP** |
| **a. Ever use** |  |  |  |  |  |  |  |  |
| **Overall** | 12.7–13.4 | 8.9–9.5 | 8.8–9.4 | 7.6–8.2 | 5.4–5.8 | 2.2–2.4 | 2.4–2.7 | 3.4–3.8 |
| **Sex** |  |  |  |  |  |  |  |  |
| Boys | 14.3–15.4 | 9.9–10.8 | 10.1–11 | 8.5–9.3 | 6.0–6.6 | 2.5–2.7 | 2.8–3.3 | 3.8–4.4 |
| Girls | 10.7–11.7 | 7.6–8.4 | 7.2–8.0 | 6.5–7.3 | 4.6–5.1 | 1.9–2.2 | 1.8–2.2 | 2.8–3.3 |
| **Age** |  |  |  |  |  |  |  |  |
| ≤12 | 5.3–6.7 | 3.2–4.3 | 3.6–4.8 | 2.6–3.6 | 1.8–2.5 | 0.8–1.1 | 1.2–1.9 | 1.3–2.0 |
| 13 | 6.8–8.2 | 4.3–5.5 | 4.6–5.8 | 3.7–4.8 | 2.6–3.4 | 1.1–1.4 | 1.2–1.8 | 1.2–1.9 |
| 14 | 9.9–11.5 | 6.1–7.4 | 6.8–8.2 | 5.1–6.4 | 3.6–4.5 | 1.5–1.9 | 1.1–1.7 | 1.3–2.0 |
| 15 | 12.4–14.3 | 9.1–10.7 | 7.7–9.3 | 8.1–9.6 | 5.7–6.7 | 2.4–2.9 | 2.0–2.8 | 2.5–3.4 |
| 16 | 14.2–16.1 | 9.8–11.4 | 9.5–11.2 | 8.3–9.9 | 5.9–7.0 | 2.4–2.9 | 2.3–3.2 | 3.2–4.2 |
| 17 | 17.6–19.7 | 13.3–15.2 | 11.7–13.5 | 11.4–13.1 | 8.2–9.4 | 3.2–3.7 | 3.1–4.1 | 5.8–7.1 |
| ≥18 | 26.1–29.8 | 18.1–21.4 | 20.9–24.4 | 15.0–18.1 | 10–12.1 | 5.0–6.0 | 6.6–8.8 | 10.1–12.8 |
| **Grade** |  |  |  |  |  |  |  |  |
| 7 | 6.0–7.3 | 3.4–4.4 | 4.1–5.1 | 2.8–3.7 | 2.0–2.6 | 0.8–1.1 | 0.9–1.4 | 0.9–1.4 |
| 8 | 8.5–10.0 | 5.3–6.5 | 6.0–7.3 | 4.5–5.7 | 3.1–4.0 | 1.4–1.7 | 1.3–2.0 | 1.5–2.2 |
| 9 | 10.8–12.5 | 7.0–8.5 | 7.4–8.9 | 6.1–7.4 | 4.3–5.2 | 1.8–2.2 | 1.5–2.2 | 1.8–2.6 |
| 10 | 14.7–16.6 | 10.6–12.2 | 9.8–11.5 | 9.2–10.8 | 6.5–7.7 | 2.7–3.1 | 2.5–3.4 | 3.4–4.4 |
| 11 | 15.6–17.6 | 11.1–12.8 | 10.4–12.1 | 9.4–11.0 | 6.7–7.9 | 2.7–3.1 | 3.0–4.0 | 4.2–5.4 |
| 12 | 19.0–21.2 | 14.4–16.4 | 13.4–15.3 | 12.3–14.1 | 8.5–9.8 | 3.8–4.3 | 4.1–5.2 | 7.4–8.9 |
| **Perceived family affluence** | | |  |  |  |  |  |  |
| Relatively poor | 17.7–21.3 | 11.4–14.5 | 13.9–17.2 | 10.3–13.2 | 7.2–9.3 | 3.1–3.9 | 4.6–6.7 | 5.5–7.8 |
| Poor to average | 15.4–17.1 | 9.7–11.2 | 10.9–12.4 | 8.3–9.6 | 6.2–7.2 | 2.1–2.4 | 2.0–2.7 | 2.8–3.6 |
| Average | 10.8–11.7 | 7.6–8.4 | 7.2–7.9 | 6.5–7.2 | 4.6–5.1 | 1.9–2.1 | 1.7–2.1 | 2.6–3.1 |
| Average to rich | 11.1–13.0 | 8.5–10.3 | 7.1–8.8 | 6.8–8.4 | 4.4–5.4 | 2.4–3.0 | 2.6–3.6 | 4.0–5.3 |
| Relatively rich | 20.2–27.0 | 17.9–24.5 | 15.5–21.7 | 16.3–22.6 | 10.1–14.0 | 6.2–8.6 | 10.4–15.8 | 12.0–17.7 |
| **Parental education** | |  |  |  |  |  |  |  |
| Primary or below | 18.6–22.5 | 12.4–15.8 | 13.5–17.0 | 11.1–14.3 | 8.1–10.5 | 3.0–3.8 | 3.5–5.6 | 4.3–6.5 |
| Secondary | 13.7–14.8 | 9.0–9.9 | 9.6–10.5 | 7.8–8.6 | 5.7–6.3 | 2.1–2.3 | 1.8–2.2 | 2.8–3.3 |
| Tertiary | 9.6–10.8 | 7.7–8.8 | 6.2–7.2 | 6.3–7.3 | 3.9–4.5 | 2.4–2.8 | 2.6–3.3 | 3.7–4.6 |
| Unknown | 11.1–12.8 | 7.8–9.2 | 7.7–9.1 | 6.8–8.1 | 4.9–5.8 | 1.9–2.3 | 2.5–3.4 | 3.1–4.0 |
| **b. Current use** | |  |  |  |  |  |  |  |
| **Overall** | 3.9–4.3 | 2.8–3.2 | 3.0–3.4 | 2.3–2.7 | 1.2–1.4 | 1.1–1.3 | 1.4–1.7 | 1.7–2.0 |
| **Sex** |  |  |  |  |  |  |  |  |
| Boys | 4.3–5.0 | 3.0–3.5 | 3.3–3.9 | 2.5–3.0 | 1.3–1.6 | 1.2–1.4 | 1.5–1.9 | 1.8–2.2 |
| Girls | 3.3–3.9 | 2.4–2.9 | 2.5–3.0 | 2.0–2.4 | 1.0–1.2 | 1.0–1.2 | 1.2–1.6 | 1.5–1.9 |
| **Age** |  |  |  |  |  |  |  |  |
| ≤12 | 1.9–2.8 | 1.6–2.4 | 1.4–2.2 | 1.3–2.1 | 0.8–1.2 | 0.5–0.9 | 0.9–1.6 | 0.9–1.5 |
| 13 | 2.0–2.9 | 1.4–2.2 | 1.4–2.1 | 1.2–1.9 | 0.7–1.2 | 0.5–0.7 | 0.7–1.2 | 0.7–1.2 |
| 14 | 2.4–3.3 | 1.5–2.3 | 1.8–2.6 | 1.4–2.1 | 0.8–1.2 | 0.6–0.9 | 0.5–1.0 | 0.6–1.1 |
| 15 | 3.8–4.9 | 2.8–3.8 | 2.8–3.7 | 2.4–3.3 | 1.0–1.4 | 1.4–1.9 | 1.4–2.2 | 1.6–2.4 |
| 16 | 3.9–5.0 | 2.6–3.6 | 3.0–4.0 | 2.2–3.0 | 1.1–1.5 | 1.1–1.5 | 1.5–2.2 | 1.7–2.5 |
| 17 | 4.9–6.2 | 3.3–4.3 | 3.8–4.9 | 2.7–3.6 | 1.3–1.7 | 1.4–1.9 | 1.5–2.2 | 2.2–3.1 |
| ≥18 | 9.3–11.9 | 6.6–8.8 | 7.4–9.7 | 5.0–7.0 | 2.6–3.6 | 2.4–3.4 | 3.3–5.0 | 4.1–6.0 |
| **Grade** |  |  |  |  |  |  |  |  |
| 7 | 1.6–2.3 | 1.2–1.8 | 1.0–1.6 | 1.0–1.5 | 0.6–0.9 | 0.4–0.6 | 0.6–1.1 | 0.6–1.0 |
| 8 | 2.5–3.4 | 1.6–2.3 | 2.0–2.8 | 1.4–2.0 | 0.9–1.3 | 0.5–0.7 | 0.8–1.3 | 0.8–1.3 |
| 9 | 3.1–4.1 | 2.1–2.9 | 2.4–3.3 | 1.7–2.4 | 0.9–1.3 | 0.8–1.1 | 0.7–1.2 | 0.8–1.4 |
| 10 | 4.6–5.7 | 3.2–4.2 | 3.5–4.6 | 2.8–3.7 | 1.3–1.7 | 1.5–2.0 | 1.7–2.4 | 1.9–2.7 |
| 11 | 4.4–5.5 | 3.2–4.2 | 3.2–4.3 | 2.6–3.6 | 1.3–1.9 | 1.3–1.7 | 1.8–2.5 | 2.2–3.1 |
| 12 | 6.0–7.3 | 4.3–5.5 | 4.7–5.9 | 3.5–4.6 | 1.6–2.1 | 1.9–2.5 | 2.2–3.0 | 2.9–3.9 |
| **Perceived family affluence** | | |  |  |  |  |  |  |
| Relatively poor | 6.0–8.3 | 4.4–6.4 | 4.7–6.8 | 3.8–5.7 | 2.1–3.1 | 1.7–2.6 | 3.0–4.7 | 3.0–4.8 |
| Poor to average | 3.0–3.9 | 2.0–2.7 | 2.5–3.2 | 1.6–2.3 | 0.8–1.2 | 0.8–1.1 | 1.0–1.5 | 1.2–1.7 |
| Average | 3.2–3.7 | 2.2–2.6 | 2.5–2.9 | 1.8–2.2 | 0.9–1.1 | 0.9–1.1 | 1.0–1.3 | 1.2–1.5 |
| Average to rich | 4.5–5.9 | 3.5–4.8 | 3.0–4.1 | 2.8–3.9 | 1.4–2.0 | 1.4–1.9 | 1.5–2.4 | 2.0–2.9 |
| Relatively rich | 13.0–18.9 | 11.8–17.4 | 10.8–16.2 | 10–15.3 | 5.2–8.0 | 4.8–7.3 | 7.0–11.7 | 8.0–12.9 |
| **Parental education** | |  |  |  |  |  |  |  |
| Primary or below | 5.1–7.5 | 3.8–5.9 | 3.9–6.1 | 3.0–5.0 | 1.8–3.0 | 1.2–2.0 | 2.0–3.7 | 2.3–4.0 |
| Secondary | 3.3–3.8 | 2.1–2.5 | 2.6–3.1 | 1.7–2.1 | 0.9–1.2 | 0.8–0.9 | 0.9–1.3 | 1.2–1.5 |
| Tertiary | 4.1–5.0 | 3.5–4.3 | 2.8–3.5 | 3.0–3.7 | 1.3–1.6 | 1.7–2.1 | 1.7–2.3 | 2.0–2.7 |
| Unknown | 4.0–5.0 | 2.6–3.5 | 3.2–4.2 | 2.2–3.0 | 1.2–1.6 | 1.0–1.4 | 1.5–2.2 | 1.7–2.4 |
| **c. Current-ever use ratio** | |  |  |  |  |  |  |  |
| **Overall** | 0.30–0.33 | 0.31–0.34 | 0.33–0.36 | 0.30–0.33 | 0.21–0.24 | 0.48–0.55 | 0.57–0.64 | 0.48–0.54 |
| **Sex** |  |  |  |  |  |  |  |  |
| Boys | 0.29–0.33 | 0.29–0.34 | 0.31–0.36 | 0.29–0.34 | 0.21–0.26 | 0.46–0.55 | 0.51–0.60 | 0.43–0.51 |
| Girls | 0.30–0.34 | 0.31–0.36 | 0.34–0.39 | 0.29–0.34 | 0.19–0.25 | 0.48–0.59 | 0.63–0.73 | 0.52–0.61 |
| **Age** |  |  |  |  |  |  |  |  |
| ≤12 | 0.32–0.43 | 0.43–0.59 | 0.34–0.49 | 0.45–0.62 | 0.33–0.54 | 0.57–0.85 | 0.66–0.87 | 0.62–0.83 |
| 13 | 0.28–0.37 | 0.31–0.43 | 0.27–0.38 | 0.29–0.42 | 0.23–0.38 | 0.37–0.61 | 0.49–0.71 | 0.48–0.70 |
| 14 | 0.23–0.31 | 0.23–0.33 | 0.25–0.34 | 0.24–0.34 | 0.18–0.29 | 0.31–0.52 | 0.43–0.67 | 0.39–0.60 |
| 15 | 0.29–0.36 | 0.29–0.37 | 0.33–0.43 | 0.28–0.37 | 0.16–0.25 | 0.52–0.69 | 0.65–0.81 | 0.60–0.76 |
| 16 | 0.26–0.32 | 0.26–0.33 | 0.30–0.38 | 0.25–0.33 | 0.16–0.24 | 0.42–0.58 | 0.58–0.73 | 0.48–0.62 |
| 17 | 0.26–0.32 | 0.23–0.29 | 0.30–0.37 | 0.22–0.29 | 0.13–0.20 | 0.41–0.56 | 0.41–0.56 | 0.35–0.45 |
| ≥18 | 0.34–0.42 | 0.34–0.44 | 0.33–0.42 | 0.31–0.41 | 0.22–0.34 | 0.43–0.61 | 0.46–0.61 | 0.38–0.50 |
| **Grade** |  |  |  |  |  |  |  |  |
| 7 | 0.25–0.34 | 0.32–0.45 | 0.22–0.33 | 0.31–0.46 | 0.24–0.41 | 0.38–0.64 | 0.60–0.82 | 0.57–0.80 |
| 8 | 0.27–0.36 | 0.28–0.38 | 0.30–0.40 | 0.27–0.38 | 0.23–0.36 | 0.30–0.51 | 0.50–0.70 | 0.43–0.63 |
| 9 | 0.28–0.35 | 0.28–0.37 | 0.31–0.39 | 0.25–0.35 | 0.18–0.28 | 0.37–0.56 | 0.41–0.62 | 0.40–0.58 |
| 10 | 0.30–0.36 | 0.28–0.36 | 0.34–0.42 | 0.28–0.36 | 0.17–0.25 | 0.51–0.67 | 0.61–0.76 | 0.52–0.66 |
| 11 | 0.27–0.33 | 0.27–0.34 | 0.29–0.37 | 0.26–0.34 | 0.18–0.26 | 0.43–0.59 | 0.52–0.67 | 0.49–0.61 |
| 12 | 0.30–0.36 | 0.28–0.35 | 0.33–0.40 | 0.27–0.34 | 0.16–0.23 | 0.48–0.61 | 0.49–0.61 | 0.37–0.46 |
| **Perceived family affluence** | | |  |  |  |  |  |  |
| Relatively poor | 0.31–0.41 | 0.34–0.47 | 0.30–0.41 | 0.33–0.46 | 0.23–0.38 | 0.47–0.72 | 0.54–0.73 | 0.47–0.65 |
| Poor to average | 0.19–0.24 | 0.19–0.25 | 0.21–0.27 | 0.18–0.24 | 0.12–0.18 | 0.32–0.48 | 0.43–0.58 | 0.38–0.51 |
| Average | 0.29–0.33 | 0.27–0.32 | 0.33–0.38 | 0.26–0.31 | 0.18–0.23 | 0.44–0.54 | 0.55–0.65 | 0.44–0.53 |
| Average to rich | 0.39–0.48 | 0.39–0.49 | 0.39–0.50 | 0.38–0.50 | 0.28–0.41 | 0.52–0.71 | 0.53–0.71 | 0.45–0.60 |
| Relatively rich | 0.59–0.75 | 0.60–0.76 | 0.63–0.80 | 0.55–0.73 | 0.42–0.66 | 0.66–0.90 | 0.59–0.80 | 0.58–0.78 |
| **Parental education** | |  |  |  |  |  |  |  |
| Primary or below | 0.25–0.35 | 0.27–0.39 | 0.25–0.37 | 0.24–0.37 | 0.17–0.31 | 0.33–0.59 | 0.46–0.69 | 0.45–0.66 |
| Secondary | 0.23–0.27 | 0.22–0.26 | 0.26–0.30 | 0.20–0.25 | 0.15–0.19 | 0.33–0.44 | 0.48–0.59 | 0.39–0.48 |
| Tertiary | 0.41–0.48 | 0.43–0.50 | 0.43–0.51 | 0.45–0.53 | 0.29–0.39 | 0.66–0.78 | 0.61–0.72 | 0.51–0.61 |
| Unknown | 0.34–0.41 | 0.31–0.39 | 0.39–0.48 | 0.29–0.38 | 0.21–0.31 | 0.46–0.63 | 0.55–0.70 | 0.48–0.62 |
| Abbreviations: TP, tobacco product; Alt TP, alternative tobacco product; Cig, cigarette; EC, e-cigarette; N-Nct, non-nicotine; Nct, nicotine; HTP, heated tobacco product; WP, waterpipe. | | | | | | | | |
| Notes: All confidence intervals were weighted by age, sex, and grade distribution of the target population provided by the Education Bureau of the Government of the Hong Kong Special Administrative Region. | | | | | | | | |

| Supplementary Table S2. Associations between sociodemographic factors and tobacco use in Hong Kong secondary school students by sex | | | | |
| --- | --- | --- | --- | --- |
|  | **Boys** | | | |
|  | **Adjusted OR (95% CI)^a^** | | | |
|  | **Cigarette** | **EC** | **HTP** | **Waterpipe** |
| a) Ever (vs never) use in all subjects | | |  |  |
| Grade |  |  |  |  |
| 7 | 1 | 1 | 1 | 1 |
| 8 | 1.47 (1.21, 1.79)^***^ | 1.45 (1.16, 1.81)^***^ | 1.32 (0.92, 1.87) | 1.34 (0.95, 1.91) |
| 9 | 1.95 (1.61, 2.35)^***^ | 1.89 (1.52, 2.33)^***^ | 1.59 (1.12, 2.24)^**^ | 1.80 (1.29, 2.52)^***^ |
| 10 | 2.48 (2.06, 3.00)^***^ | 2.80 (2.28, 3.46)^***^ | 2.30 (1.65, 3.19)^***^ | 3.04 (2.20, 4.18)^***^ |
| 11 | 2.30 (1.89, 2.79)^***^ | 2.64 (2.13, 3.27)^***^ | 2.65 (1.91, 3.70)^***^ | 3.53 (2.56, 4.86)^***^ |
| 12 | 3.12 (2.49, 3.90)^***^ | 3.72 (2.92, 4.76)^***^ | 3.30 (2.26, 4.84)^***^ | 5.88 (4.13, 8.37)^***^ |
| P for trend | <0.001**^†^** | <0.001**^†^** | <0.001**^†^** | <0.001**^†^** |
| Perceived family affluence | |  |  |  |
| Relatively poor | 1 | 1 | 1 | 1 |
| Poor to average | 0.79 (0.66, 0.94)^**^ | 0.84 (0.69, 1.03) | 0.42 (0.32, 0.56)^***^ | 0.46 (0.35, 0.60)^***^ |
| Average | 0.60 (0.51, 0.71)^***^ | 0.78 (0.65, 0.94)^*^ | 0.42 (0.32, 0.54)^***^ | 0.47 (0.37, 0.60)^***^ |
| Average to rich | 0.69 (0.55, 0.86)^***^ | 0.83 (0.65, 1.05) | 0.65 (0.46, 0.91)^*^ | 0.76 (0.56, 1.03) |
| Relatively rich | 1.52 (1.11, 2.07)^**^ | 2.10 (1.52, 2.90)^***^ | 2.05 (1.36, 3.10)^***^ | 2.22 (1.50, 3.28)^***^ |
| P for trend | <0.001^‡^ | <0.001^‡^ | <0.001^‡^ | <0.001^‡^ |
| Parental education | |  |  |  |
| Primary or below | 1 | 1 | 1 | 1 |
| Secondary | 0.84 (0.70, 1.00) | 0.84 (0.69, 1.03) | 0.59 (0.44, 0.79)^***^ | 0.63 (0.48, 0.83)^**^ |
| Tertiary | 0.61 (0.49, 0.75)^***^ | 0.73 (0.57, 0.92)^**^ | 0.64 (0.46, 0.90)^*^ | 0.66 (0.48, 0.90)^**^ |
| P for trend | <0.001**^†^** | <0.01**^†^** | <0.001^‡^ | <0.01^‡^ |
| b) Current (vs non-current) use in all subjects | | | |  |
| Grade |  |  |  |  |
| 7 | 1 | 1 | 1 | 1 |
| 8 | 1.74 (1.23, 2.47)^**^ | 1.18 (0.82, 1.69) | 1.00 (0.64, 1.57) | 1.07 (0.68, 1.67) |
| 9 | 2.67 (1.92, 3.72)^***^ | 1.58 (1.12, 2.23)^**^ | 1.35 (0.89, 2.07) | 1.45 (0.95, 2.21) |
| 10 | 3.65 (2.63, 5.05)^***^ | 2.18 (1.56, 3.05)^***^ | 2.09 (1.40, 3.12)^***^ | 2.43 (1.63, 3.61)^***^ |
| 11 | 2.98 (2.13, 4.18)^***^ | 2.16 (1.53, 3.05)^***^ | 2.44 (1.63, 3.65)^***^ | 2.80 (1.88, 4.18)^***^ |
| 12 | 3.78 (2.58, 5.55)^***^ | 2.27 (1.51, 3.41)^***^ | 1.95 (1.18, 3.22)^**^ | 2.77 (1.71, 4.47)^***^ |
| P for trend | <0.001**^†^** | <0.001**^†^** | <0.001**^†^** | <0.001**^†^** |
| Perceived family affluence | |  |  |  |
| Relatively poor | 1 | 1 | 1 | 1 |
| Poor to average | 0.53 (0.40, 0.71)^***^ | 0.41 (0.30, 0.56)^***^ | 0.28 (0.19, 0.40)^***^ | 0.33 (0.23, 0.48)^***^ |
| Average | 0.66 (0.51, 0.85)^**^ | 0.48 (0.36, 0.63)^***^ | 0.35 (0.25, 0.47)^***^ | 0.39 (0.29, 0.53)^***^ |
| Average to rich | 0.96 (0.69, 1.32) | 0.72 (0.50, 1.02) | 0.58 (0.39, 0.88)^*^ | 0.67 (0.45, 1.00)^*^ |
| Relatively rich | 3.00 (2.01, 4.49)^***^ | 2.16 (1.41, 3.33)^***^ | 2.08 (1.29, 3.37)^**^ | 2.20 (1.36, 3.54)^**^ |
| P for trend | <0.001^‡^ | <0.001^‡^ | <0.001^‡^ | <0.001^‡^ |
| Parental education | |  |  |  |
| Primary or below | 1 | 1 | 1 | 1 |
| Secondary | 0.61 (0.46, 0.81)^***^ | 0.56 (0.41, 0.76)^***^ | 0.42 (0.29, 0.59)^***^ | 0.44 (0.31, 0.62)^***^ |
| Tertiary | 0.56 (0.41, 0.77)^***^ | 0.63 (0.44, 0.90)^*^ | 0.43 (0.29, 0.65)^***^ | 0.48 (0.33, 0.72)^***^ |
| P for trend | <0.01**^†^** | <0.001^‡^ | <0.001^‡^ | <0.001^‡^ |
| c) Current (vs non-current) use in ever users | | | |  |
| Grade |  |  |  |  |
| 7 | 1 | 1 | 1 | 1 |
| 8 | 1.14 (0.71, 1.82) | 0.74 (0.45, 1.21) | 0.74 (0.35, 1.57) | 0.69 (0.33, 1.48) |
| 9 | 1.41 (0.91, 2.20) | 0.81 (0.51, 1.28) | 0.74 (0.36, 1.53) | 0.74 (0.36, 1.49) |
| 10 | 1.88 (1.22, 2.90)^**^ | 0.82 (0.52, 1.29) | 1.00 (0.50, 2.00) | 0.90 (0.46, 1.76) |
| 11 | 1.63 (1.05, 2.55)^*^ | 0.91 (0.57, 1.44) | 1.14 (0.57, 2.27) | 0.84 (0.43, 1.64) |
| 12 | 1.68 (1.02, 2.75)^*^ | 0.59 (0.35, 1.01) | 0.40 (0.18, 0.87)^*^ | 0.40 (0.19, 0.82)^*^ |
| P for trend | 0.03**^†^** | 0.65**^†^** | 0.26**^†^** | 0.05**^†^** |
| Perceived family affluence | |  |  |  |
| Relatively poor | 1 | 1 | 1 | 1 |
| Poor to average | 0.65 (0.45, 0.94)^*^ | 0.38 (0.25, 0.58)^***^ | 0.35 (0.19, 0.65)^***^ | 0.46 (0.26, 0.81)^**^ |
| Average | 1.09 (0.77, 1.53) | 0.52 (0.36, 0.76)^***^ | 0.63 (0.36, 1.09) | 0.81 (0.49, 1.34) |
| Average to rich | 1.93 (1.25, 2.99)^**^ | 0.95 (0.59, 1.54) | 0.86 (0.43, 1.70) | 0.96 (0.52, 1.78) |
| Relatively rich | 3.85 (2.15, 6.90)^***^ | 1.37 (0.74, 2.51) | 1.24 (0.54, 2.86) | 1.19 (0.56, 2.52) |
| P for trend | <0.001^‡^ | <0.01^‡^ | 0.01^‡^ | 0.41^‡^ |
| Parental education | |  |  |  |
| Primary or below | 1 | 1 | 1 | 1 |
| Secondary | 0.52 (0.36, 0.74)^***^ | 0.55 (0.36, 0.84)^**^ | 0.57 (0.30, 1.11) | 0.51 (0.29, 0.90)^*^ |
| Tertiary | 0.85 (0.56, 1.28) | 0.85 (0.53, 1.35) | 0.52 (0.25, 1.05) | 0.53 (0.28, 0.99)^*^ |
| P for trend | <0.001^‡^ | <0.001^‡^ | 0.16**^†^** | 0.05^‡^ |
|  | **Girls** | | | |
|  | **Adjusted OR (95% CI)^a^** | | | |
|  | **Cigarette** | **EC** | **HTP** | **Waterpipe** |
| a) Ever (vs never) use in all subjects | | |  |  |
| Grade |  |  |  |  |
| 7 | 1 | 1 | 1 | 1 |
| 8 | 1.50 (1.18, 1.91)^***^ | 1.79 (1.34, 2.40)^***^ | 1.51 (0.91, 2.50) | 1.96 (1.21, 3.18)^**^ |
| 9 | 1.61 (1.27, 2.04)^***^ | 2.46 (1.86, 3.25)^***^ | 1.65 (1.00, 2.70)^*^ | 2.20 (1.37, 3.54)^**^ |
| 10 | 2.04 (1.62, 2.58)^***^ | 3.56 (2.72, 4.68)^***^ | 2.38 (1.49, 3.82)^***^ | 3.55 (2.26, 5.60)^***^ |
| 11 | 2.37 (1.88, 3.00)^***^ | 3.68 (2.79, 4.85)^***^ | 2.84 (1.77, 4.55)^***^ | 4.50 (2.86, 7.08)^***^ |
| 12 | 2.84 (2.14, 3.76)^***^ | 4.24 (3.09, 5.81)^***^ | 4.34 (2.57, 7.32)^***^ | 8.93 (5.49, 14.50)^***^ |
| P for trend | <0.001**^†^** | <0.001**^†^** | <0.001**^†^** | <0.001**^†^** |
| Perceived family affluence | |  |  |  |
| Relatively poor | 1 | 1 | 1 | 1 |
| Poor to average | 0.74 (0.57, 0.96)^*^ | 0.65 (0.49, 0.86)^**^ | 0.35 (0.23, 0.55)^***^ | 0.40 (0.27, 0.60)^***^ |
| Average | 0.60 (0.47, 0.77)^***^ | 0.60 (0.46, 0.79)^***^ | 0.35 (0.23, 0.52)^***^ | 0.40 (0.28, 0.58)^***^ |
| Average to rich | 0.89 (0.66, 1.20) | 1.02 (0.74, 1.39) | 0.74 (0.46, 1.20) | 0.78 (0.51, 1.21) |
| Relatively rich | 1.85 (1.20, 2.86)^**^ | 2.43 (1.57, 3.76)^***^ | 3.19 (1.82, 5.60)^***^ | 3.17 (1.86, 5.37)^***^ |
| P for trend | <0.001^‡^ | <0.001^‡^ | <0.001^‡^ | <0.001^‡^ |
| Parental education | |  |  |  |
| Primary or below | 1 | 1 | 1 | 1 |
| Secondary | 0.80 (0.63, 1.02) | 0.72 (0.56, 0.93)^*^ | 0.65 (0.41, 1.04) | 0.86 (0.56, 1.31) |
| Tertiary | 0.66 (0.50, 0.88)^**^ | 0.64 (0.48, 0.86)^**^ | 1.14 (0.69, 1.88) | 1.05 (0.66, 1.67) |
| P for trend | 0.02**^†^** | 0.05**^†^** | <0.001^‡^ | 0.10^‡^ |
| b) Current (vs non-current) use in all subjects | | | |  |
| Grade |  |  |  |  |
| 7 | 1 | 1 | 1 | 1 |
| 8 | 1.89 (1.28, 2.78)^**^ | 1.65 (1.04, 2.61)^*^ | 1.57 (0.87, 2.83) | 1.58 (0.86, 2.90) |
| 9 | 1.64 (1.10, 2.43)^*^ | 1.62 (1.02, 2.56)^*^ | 1.16 (0.62, 2.16) | 1.67 (0.92, 3.04) |
| 10 | 2.09 (1.43, 3.07)^***^ | 2.29 (1.48, 3.56)^***^ | 2.38 (1.36, 4.14)^**^ | 2.86 (1.63, 5.01)^***^ |
| 11 | 2.02 (1.36, 3.00)^***^ | 1.98 (1.25, 3.14)^**^ | 1.84 (1.02, 3.31)^*^ | 2.99 (1.69, 5.28)^***^ |
| 12 | 2.84 (1.82, 4.43)^***^ | 3.11 (1.88, 5.16)^***^ | 3.98 (2.13, 7.44)^***^ | 5.99 (3.27, 10.99)^***^ |
| P for trend | <0.001**^†^** | <0.001**^†^** | <0.001**^†^** | <0.001**^†^** |
| Perceived family affluence | |  |  |  |
| Relatively poor | 1 | 1 | 1 | 1 |
| Poor to average | 0.50 (0.34, 0.75)^***^ | 0.37 (0.24, 0.60)^***^ | 0.36 (0.20, 0.62)^***^ | 0.36 (0.21, 0.60)^***^ |
| Average | 0.48 (0.33, 0.69)^***^ | 0.42 (0.28, 0.64)^***^ | 0.30 (0.18, 0.49)^***^ | 0.31 (0.20, 0.50)^***^ |
| Average to rich | 0.81 (0.52, 1.25) | 0.86 (0.53, 1.39) | 0.71 (0.39, 1.27) | 0.69 (0.40, 1.20) |
| Relatively rich | 2.97 (1.74, 5.06)^***^ | 3.62 (2.06, 6.37)^***^ | 4.09 (2.15, 7.77)^***^ | 4.32 (2.36, 7.91)^***^ |
| P for trend | <0.001^‡^ | <0.001^‡^ | <0.001^‡^ | <0.001^‡^ |
| Parental education | |  |  |  |
| Primary or below | 1 | 1 | 1 | 1 |
| Secondary | 0.78 (0.52, 1.16) | 0.48 (0.32, 0.74)^***^ | 0.44 (0.25, 0.76)^**^ | 0.59 (0.35, 0.99)^*^ |
| Tertiary | 0.95 (0.61, 1.47) | 0.80 (0.50, 1.26) | 1.05 (0.59, 1.87) | 1.06 (0.61, 1.86) |
| P for trend | 0.03^‡^ | <0.001^‡^ | <0.001^‡^ | <0.001^‡^ |
| c) Current (vs non-current) use in ever users | | | |  |
| Grade |  |  |  |  |
| 7 | 1 | 1 | 1 | 1 |
| 8 | 1.25 (0.70, 2.26) | 0.89 (0.45, 1.75) | 0.58 (0.17, 1.94) | 0.38 (0.12, 1.23) |
| 9 | 1.60 (0.90, 2.83) | 0.82 (0.43, 1.56) | 0.44 (0.14, 1.40) | 0.51 (0.16, 1.63) |
| 10 | 1.82 (1.05, 3.15)^*^ | 0.77 (0.41, 1.44) | 1.19 (0.39, 3.64) | 0.58 (0.20, 1.74) |
| 11 | 1.43 (0.82, 2.50) | 0.73 (0.39, 1.38) | 0.37 (0.13, 1.11) | 0.43 (0.14, 1.29) |
| 12 | 1.98 (1.06, 3.68)^*^ | 1.10 (0.55, 2.18) | 1.34 (0.41, 4.33) | 0.69 (0.23, 2.07) |
| P for trend | 0.21**^†^** | 0.65**^†^** | 0.59**^†^** | 0.75**^†^** |
| Perceived family affluence | |  |  |  |
| Relatively poor | 1 | 1 | 1 | 1 |
| Poor to average | 0.45 (0.26, 0.79)^**^ | 0.35 (0.19, 0.66)^**^ | 0.72 (0.24, 2.22) | 0.78 (0.30, 2.01) |
| Average | 0.57 (0.35, 0.95)^*^ | 0.52 (0.29, 0.92)^*^ | 0.62 (0.22, 1.71) | 0.48 (0.20, 1.13) |
| Average to rich | 0.81 (0.45, 1.48) | 1.00 (0.52, 1.90) | 0.93 (0.29, 3.00) | 0.73 (0.28, 1.91) |
| Relatively rich | 6.03 (2.34, 15.56)^***^ | 4.16 (1.61, 10.76)^**^ | 8.30 (1.44, 47.99)^*^ | 9.28 (1.74, 49.33)^**^ |
| P for trend | <0.001^‡^ | <0.001^‡^ | <0.01^‡^ | <0.001^‡^ |
| Parental education | |  |  |  |
| Primary or below | 1 | 1 | 1 | 1 |
| Secondary | 0.98 (0.57, 1.70) | 0.61 (0.34, 1.09) | 0.35 (0.10, 1.22) | 0.61 (0.21, 1.76) |
| Tertiary | 2.20 (1.21, 4.01)^**^ | 1.72 (0.92, 3.21) | 0.94 (0.26, 3.43) | 1.37 (0.45, 4.12) |
| P for trend | 0.01^‡^ | <0.001^‡^ | 0.01^‡^ | 0.06^‡^ |
| ^*^ P < 0.05, ^**^ P < 0.01, ^***^ P < 0.001. ^†^ Linear trend. ^‡^ Curvilinear (quadratic) trend. ^§^ Interaction with sex. Abbreviations: EC, e-cigarette; HTP, heated tobacco product; OR, odds ratio; CI, confidence interval. ^a^ Adjusted odds ratios adjusted for sex, grade, perceived family affluence, parental education and school clustering effects. | | | | |

| **Supplementary Table S3. Current-ever use ratios of various tobacco products in Hong Kong secondary school students by sociodemographic factors, excluding experimenters of the respective products** | | | | | |
| --- | --- | --- | --- | --- | --- |
|  | **Cig** | **EC** | **HTP** | **WP** |  |
| **Overall** | 0.77 | 0.66 | 0.88 | 0.84 |  |
| **Sex** |  |  |  |  |  |
| Boys | 0.76 | 0.64 | 0.55 | 0.78 |  |
| Girls | 0.79 | 0.71 | 0.94 | 0.92 |  |
| **Age** |  |  |  |  |  |
| ≤12 | - | 0.95 | - | 0.99 |  |
| 13 | 0.82 | 0.77 | 0.79 | 0.91 |  |
| 14 | 0.71 | 0.59 | 0.93 | 0.90 |  |
| 15 | 0.74 | 0.68 | 0.95 | 0.99 |  |
| 16 | 0.82 | 0.66 | 0.95 | 0.86 |  |
| 17 | 0.75 | 0.60 | 0.75 | 0.71 |  |
| ≥18 | 0.73 | 0.62 | 0.83 | 0.74 |  |
| **Grade** |  |  |  |  |  |
| 7 | 0.80 | 0.78 | 0.98 | 0.93 |  |
| 8 | 0.81 | 0.66 | 0.91 | 0.88 |  |
| 9 | 0.72 | 0.57 | 0.88 | 0.87 |  |
| 10 | 0.75 | 0.72 | 0.95 | 0.85 |  |
| 11 | 0.80 | 0.70 | 0.88 | 0.96 |  |
| 12 | 0.76 | 0.62 | 0.79 | 0.71 |  |
| **Perceived family affluence** |  |  |  |  |  |
| Relatively poor | 0.80 | 0.70 | 0.97 | 0.81 |  |
| Poor to average | 0.69 | 0.58 | 0.78 | 0.79 |  |
| Average | 0.77 | 0.65 | 0.90 | 0.83 |  |
| Average to rich | 0.77 | 0.70 | 0.83 | 0.86 |  |
| Relatively rich | 0.91 | 0.81 | 0.91 | 0.93 |  |
| **Parental education** |  |  |  |  |  |
| Primary or below | 0.72 | 0.70 | 0.96 | 0.85 |  |
| Secondary | 0.72 | 0.53 | 0.82 | 0.78 |  |
| Tertiary | 0.82 | 0.82 | 0.90 | 0.86 |  |
| Unknown | 0.83 | 0.66 | 0.88 | 0.87 |  |
| Abbreviations: TP, tobacco product; Alt TP, alternative tobacco product; Cig, cigarette; EC, e-cigarette; N-Nct, non-nicotine; Nct, nicotine; HTP, heated tobacco product; WP, waterpipe. | | | | | |
| Notes: All percentages were weighted by age, sex, and grade distribution of the target population provided by the Education Bureau of the Government of the Hong Kong Special Administrative Region. A few figures were not shown due to insufficient cases in the subgroups. | | | | | |

| Supplementary Table S4. Associations between sociodemographic factors and tobacco use in Hong Kong secondary school students, using never users as the reference group | | | | |
| --- | --- | --- | --- | --- |
|  | **Adjusted OR (95% CI)^a^** | | | |
|  | **Cigarette** | **EC** | **HTP** | **Waterpipe** |
| a) Ever (vs never) use | |  |  |  |
| Sex |  |  |  |  |
| Boys | 1 | 1 | 1 | 1 |
| Girls | 0.78 (0.71, 0.84)^***^ | 0.84 (0.77, 0.92)^***^ | 0.73 (0.63, 0.85)^***^ | 0.78 (0.68, 0.89)^***^ |
| Grade |  |  |  |  |
| 7 | 1 | 1 | 1 | 1 |
| 8 | 1.48 (1.27, 1.72)^***^ | 1.57 (1.32, 1.88)^***^ | 1.42 (1.06, 1.90)^*^ | 1.60 (1.20, 2.13)^**^ |
| 9 | 1.82 (1.57, 2.11)^***^ | 2.10 (1.77, 2.49)^***^ | 1.71 (1.28, 2.27)^***^ | 2.05 (1.55, 2.70)^***^ |
| 10 | 2.34 (2.02, 2.71)^***^ | 3.11 (2.63, 3.67)^***^ | 2.47 (1.88, 3.25)^***^ | 3.40 (2.61, 4.43)^***^ |
| 11 | 2.36 (2.04, 2.75)^***^ | 3.07 (2.59, 3.64)^***^ | 2.88 (2.19, 3.80)^***^ | 4.04 (3.10, 5.26)^***^ |
| 12 | 3.05 (2.55, 3.64)^***^ | 4.00 (3.29, 4.87)^***^ | 3.94 (2.88, 5.38)^***^ | 7.23 (5.41, 9.66)^***^ |
| Perceived family affluence | |  |  |  |
| Relatively poor | 1 | 1 | 1 | 1 |
| Poor to average | 0.78 (0.67, 0.90)^***^ | 0.76 (0.65, 0.90)^**^ | 0.41 (0.32, 0.52)^***^ | 0.45 (0.36, 0.56)^***^ |
| Average | 0.61 (0.53, 0.70)^***^ | 0.69 (0.59, 0.80)^***^ | 0.39 (0.31, 0.48)^***^ | 0.44 (0.35, 0.53)^***^ |
| Average to rich | 0.77 (0.64, 0.92)^**^ | 0.88 (0.73, 1.07) | 0.67 (0.51, 0.88)^**^ | 0.74 (0.57, 0.95)^*^ |
| Relatively rich | 1.73 (1.34, 2.22)^***^ | 2.18 (1.68, 2.83)^***^ | 2.62 (1.88, 3.64)^***^ | 2.62 (1.91, 3.59)^***^ |
| Parental education | |  |  |  |
| Primary or below | 1 | 1 | 1 | 1 |
| Secondary | 0.82 (0.71, 0.95)^**^ | 0.80 (0.68, 0.94)^**^ | 0.61 (0.47, 0.78)^***^ | 0.70 (0.55, 0.88)^**^ |
| Tertiary | 0.63 (0.53, 0.75)^***^ | 0.68 (0.57, 0.82)^***^ | 0.77 (0.58, 1.02) | 0.75 (0.57, 0.97)^*^ |
| b) Current (vs never) use | | |  |  |
| Sex |  |  |  |  |
| Boys | 1 | 1 | 1 | 1 |
| Girls | 0.84 (0.74, 0.96)^*^ | 0.86 (0.73, 1.00)^*^ | 0.88 (0.73, 1.06) | 0.93 (0.78, 1.10) |
| Grade |  |  |  |  |
| 7 | 1 | 1 | 1 | 1 |
| 8 | 1.84 (1.42, 2.40)^***^ | 1.39 (1.04, 1.85)^*^ | 1.24 (0.87, 1.77) | 1.31 (0.92, 1.87) |
| 9 | 2.29 (1.78, 2.96)^***^ | 1.67 (1.26, 2.21)^***^ | 1.40 (0.99, 1.97) | 1.66 (1.18, 2.34)^**^ |
| 10 | 3.11 (2.43, 4.00)^***^ | 2.35 (1.79, 3.09)^***^ | 2.37 (1.71, 3.27)^***^ | 2.78 (2.01, 3.84)^***^ |
| 11 | 2.70 (2.08, 3.50)^***^ | 2.24 (1.70, 2.97)^***^ | 2.39 (1.72, 3.33)^***^ | 3.07 (2.21, 4.25)^***^ |
| 12 | 3.73 (2.78, 5.01)^***^ | 2.90 (2.10, 4.00)^***^ | 2.98 (2.02, 4.40)^***^ | 4.38 (3.02, 6.36)^***^ |
| Perceived family affluence | |  |  |  |
| Relatively poor | 1 | 1 | 1 | 1 |
| Poor to average | 0.52 (0.41, 0.66)^***^ | 0.40 (0.31, 0.52)^***^ | 0.30 (0.22, 0.41)^***^ | 0.35 (0.26, 0.46)^***^ |
| Average | 0.56 (0.45, 0.69)^***^ | 0.44 (0.35, 0.55)^***^ | 0.32 (0.24, 0.41)^***^ | 0.35 (0.27, 0.45)^***^ |
| Average to rich | 0.85 (0.65, 1.11) | 0.74 (0.56, 0.98)^*^ | 0.61 (0.44, 0.85)^**^ | 0.65 (0.47, 0.90)^**^ |
| Relatively rich | 3.06 (2.22, 4.22)^***^ | 2.78 (1.98, 3.91)^***^ | 2.99 (2.07, 4.34)^***^ | 3.25 (2.26, 4.67)^***^ |
| Parental education | |  |  |  |
| Primary or below | 1 | 1 | 1 | 1 |
| Secondary | 0.66 (0.52, 0.83)^***^ | 0.53 (0.41, 0.69)^***^ | 0.43 (0.32, 0.58)^***^ | 0.49 (0.36, 0.65)^***^ |
| Tertiary | 0.66 (0.51, 0.86)^**^ | 0.67 (0.50, 0.89)^**^ | 0.61 (0.44, 0.85)^**^ | 0.65 (0.47, 0.90)^**^ |
| ^*^ P < 0.05, ^**^ P < 0.01, ^***^ P < 0.001. Abbreviations: EC, e-cigarette; HTP, heated tobacco product; OR, odds ratio; CI, confidence interval. ^a^ Adjusted odds ratios adjusted for sex, grade, perceived family affluence, parental education and school clustering effects. | | | | |
